# Emergence of a carbapenem-resistant *Klebsiella pneumoniae* ST15-KL19 clone with elevated virulence potential in a tertiary hospital in China

**DOI:** 10.3389/fcimb.2026.1745492

**Published:** 2026-04-07

**Authors:** Guiling Li, Yujing Zhang, Guqi Ren, Longlong Tian, Xinan Jiao, Lin Sun, Chuanli Ren

**Affiliations:** 1 Clinical Testing Center, Northern Jiangsu People’s Hospital Affiliated to Yangzhou University, Yangzhou, China; 2 Jiangsu Key Laboratory of Zoonosis, Yangzhou University, Yangzhou, China; 3 Jiangsu Co-Innovation Center for Prevention and Control of Important Animal Infectious Diseases and Zoonoses, Yangzhou University, Yangzhou, China; 4 Key Laboratory of Prevention and Control of Biological Hazard Factors (Animal Origin) for Agrifood Safety and Quality, Ministry of Agriculture and Rural Affairs of China, Yangzhou University, Yangzhou, China

**Keywords:** carbapenem-resistant, KL19, *Klebsiella pneumoniae*, ST15, virulence potential

## Abstract

**Introduction:**

Carbapenem-resistant *Klebsiella pneumoniae* (CRKP) has become a significant global health concern. The convergence of carbapenem resistance and elevated virulence potential has led to increasingly difficult-to-treat *K. pneumoniae* strains. Among these, ST15 is an expanding global high-risk clone, yet variants with elevated virulence potential carrying the KL19 capsular type remain rarely reported.

**Methods:**

In this study, we characterized the phenotypic and genomic features of a carbapenem-resistant *K. pneumoniae* ST15-KL19 clone exhibiting elevated virulence potential, isolated from a tertiary hospital in eastern China.

**Results:**

All isolates exhibited extensive drug resistance, including resistance to carbapenems, and carried the *bla*_KPC-2_ gene. Whole-genome sequencing revealed a conserved chromosomal virulence-associated profile and hybrid plasmids carrying multiple resistance determinants. The *bla*_KPC-2_ gene was located within a stable composite transposon, suggesting efficient mobility and long-term maintenance. Phylogenetic analysis demonstrated that the isolates were closely related to other Chinese ST15-KL19 strains, indicating recent clonal expansion and local dissemination.

**Discussion:**

The identification of this clone highlights the ongoing convergence of multidrug resistance and elevated virulence potential in *K. pneumoniae*, which poses challenges for infection control and clinical management. These findings emphasize the importance of continuous genomic surveillance to monitor the spread of high-risk *K. pneumoniae* lineages.

## 1 Introduction

Carbapenem-resistant *Klebsiella pneumoniae* (CRKP) has emerged as a major global public health threat due to its extensive resistance to most β-lactam antibiotics and its ability to cause severe infections in both healthcare and community settings (van Duin and Doi, 2017). In recent years, the convergence of carbapenem resistance and hypervirulence has led to the rise of carbapenem-resistant hypervirulent *K. pneumoniae* (CR-hvKP), presenting new challenges in clinical treatment and infection control (Shon et al., 2013). Recent surveillance studies have demonstrated an increasing trend of carbapenem-resistant hypervirulent *Klebsiella pneumoniae* (CR-hvKP) in China and globally (Lei et al., 2024; Pu et al., 2023; Chen et al., 2024; Han et al., 2022). Traditionally, hypervirulent *K. pneumoniae* (hvKP) strains have been associated with sequence types ST23, ST65, and ST86, which commonly harbor capsular serotypes K1 or K2 and key virulence factors such as *rmpA*, *rmpA2*, and the aerobactin synthesis locus (*iucABCD*). These genes contribute to increased capsule production and iron acquisition, promoting a hypermucoviscous phenotype (Shon et al., 2013; Wyres et al., 2020). In contrast, carbapenem resistant is mainly linked to classical lineages such as ST11 and ST258, which typically carry plasmid-borne carbapenemase genes, including *bla*_KPC_, *bla*_NDM_, and *bla*_OXA-48_ like genes (Gu et al., 2018; David et al., 2019).

Recent genomic studies have reported the emergence of hybrid CR-hvKP strains due to horizontal gene transfer, especially plasmid-mediated acquisition of virulence and resistance genes. This convergence complicates clinical identification, antimicrobial therapy, and epidemiological surveillance (Rodrigues et al., 2023). In China, ST11 remains the dominant CR-hvKP lineage, however, ST15, an internationally disseminated sequence type, has recently drawn attention due to its increasing association with *bla*_KPC-2_ and *bla*_OXA_ carbapenemase genes (Rodrigues et al., 2023; Peirano et al., 2020). Despite this, variants with elevated virulence potential within the ST15 lineage remain exceedingly rare. Notably, capsular type KL19, which is not traditionally associated with hypervirulence, has recently been identified in clinical ST15 isolates co-harboring both virulence and carbapenem resistance determinants (Feng et al., 2023; Wang et al., 2022). These findings suggest an ongoing genomic recombination process within *K. pneumoniae* lineages under antimicrobial pressure (Tian et al., 2022).

In this study, we describe the phenotypic and genomic characteristics of a carbapenem-resistant *K. pneumoniae* ST15-KL19 clone exhibiting elevated virulence potential, isolated from patients in a tertiary hospital in eastern China. We performed antimicrobial susceptibility testing, whole-genome sequencing, and virulence assays to investigate the molecular features and evolutionary implications of this emerging high-risk lineage.

## 2 Materials and methods

### 2.1 Clinical isolates and identification

Between August and September 2023, seven non-duplicate clinical *K. pneumoniae* isolates were recovered from hospitalized patients in a tertiary-care hospital in eastern China. The isolates were obtained from routine clinical microbiological cultures performed as part of standard diagnostic workflows. All strains were cultured on Columbia blood agar supplemented with 5% sheep blood and were identified using the VITEK^®^ 2 Compact system (bioMérieux, France). These isolates were selected for further analysis based on their resistance to carbapenems, tigecycline, and colistin.

### 2.2 Antimicrobial susceptibility testing

Minimum inhibitory concentrations (MICs) for 15 antimicrobial agents were determined using the agar dilution method, except for colistin and tigecycline, which were assessed using broth microdilution. The tested antibiotics included: ampicillin, cefotaxime, meropenem, gentamicin, amikacin, streptomycin, tetracycline, tigecycline, nalidixic acid, ciprofloxacin, colistin, trimethoprim-sulfamethoxazole, chloramphenicol, florfenicol, and fosfomycin. Results were interpreted according to the Clinical and Laboratory Standards Institute (CLSI) guidelines (M100-S35, 2025 edition). Tigecycline and florfenicol breakpoints were interpreted using European Committee on Antimicrobial Susceptibility Testing (EUCAST) criteria. *Escherichia coli* ATCC 25922 was used as the quality control strain.

### 2.3 String test

Hypermucoviscosity was assessed using the string test. Overnight cultures grown on Columbia blood agar with 5% sheep blood at 37°C were examined by touching a colony with a sterile inoculation loop and lifting it vertically. A viscous string ≥5 mm in length was considered a positive result (Li et al., 2020).

### 2.4 *Galleria mellonella* infection model

Virulence potential was evaluated using a modified *G. mellonella* larval infection model (Mai et al., 2023). Overnight cultures were washed with phosphate-buffered saline (PBS) and adjusted to 1 × 10^6^ CFU/mL. Ten larvae per group were injected in the right hind proleg with 10 μL of the bacterial suspension. Control groups included larvae injected with PBS, a hypervirulent reference strain H27, and a low-virulence strain L51 (Huang et al., 2023). Following injection, larvae were incubated at 37°C in the dark, and survival was monitored over 60 hours. Each experiment was performed in triplicate, and survival curves were generated from representative results of three independent biological replicates.

### 2.5 Whole-genome sequencing and bioinformatic analysis

Genomic DNA was extracted and subjected to both short-read (Illumina NovaSeq) and long-read (Oxford Nanopore MinION) sequencing. Hybrid assemblies were generated with SPAdes v3.11 (Antipov et al., 2016) and Unicycler v0.4.9 (Wick et al., 2017), followed by sequence polishing with Pilon v1.23 (Walker et al., 2014). Genome annotation was performed using Prokka v1.13 (Seemann, 2014). Antimicrobial resistance genes and plasmid replicons were identified using tools from the Center for Genomic Epidemiology (http://www.genomicepidemiology.org/) (Zankari et al., 2012).

Virulence-associated genes were identified by comparison against the Virulence Factors Database (VFDB, http://www.mgc.ac.cn/VFs) (Chen et al., 2005), using BLASTn with a minimum identity threshold of 90% and coverage of 80%.

In silico multilocus sequence typing (MLST) was performed via the Institut Pasteur database (https://bigsdb.pasteur.fr/). Plasmid alignments were visualized using the BLAST Ring Image Generator (BRIG) (Alikhan et al., 2011), and resistance gene contexts were analyzed using Easyfig (Sullivan et al., 2011). Reference genomes were retrieved from the NCBI database. Core genome single nucleotide polymorphism (SNP)-based phylogenetic trees were reconstructed using ParSNP v1.2 (https://github.com/marbl/parsnp) (Treangen et al., 2014) and visualized with ChiPlot (http://chiplot.online/#) (Xie et al., 2023).

### 2.6 Conjugation assays

To evaluate the transferability of *bla*_KPC-2_-carrying plasmids, conjugation assays were performed using *K. pneumoniae* isolates as donors and streptomycin-resistant *E. coli* C600 as the recipient. Mating mixtures were plated onto LB agar containing streptomycin (3000 mg/L) and meropenem (2 mg/L). Transconjugants were selected on the same selective medium and confirmed by PCR amplification of *bla*_KPC-2_.

### 2.7 Plasmid stability assay

To assess plasmid stability, all *bla*_KPC-2_-positive isolates were serially passaged in antibiotic-free LB broth at a 1:1000 dilution twice daily for 20 consecutive days. At 5-day intervals, cultures were plated on non-selective LB agar and 100 colonies were randomly picked for PCR detection of *bla*_KPC-2_. Plasmid retention was calculated as the percentage of colonies positive for the gene.

### 2.8 Data availability

The genome sequences generated in this study have been deposited into CNSA with accession number CNP0007685.

## 3 Results

### 3.1 Antimicrobial susceptibility profiles

Seven non-duplicate *K. pneumoniae* isolates were recovered from hospitalized patients at a tertiary-care hospital in Jiangsu Province, China. Six isolates were obtained from sputum samples and one from urine. Five isolates originated from the Neurological Intensive Care Unit, one from the Emergency Intensive Care Unit, and one from the Department of Neurology (**Table 1**). These isolates were initially identified due to their co-resistance to meropenem, tigecycline, and colistin, three last-line antibiotics.

**Table 1:** Clinical characteristics and antimicrobial susceptibility profiles of carbapenem-resistant *K. pneumoniae* ST15-KL19 isolates.

Strain	Source	Department	ST	K locus	String test	MIC (mg/L)														
						AMP	CTX	MEM	GEN	AMK	STR	TET	TIL	CHL	FFC	NAL	CIP	CL	FOS	SXT
SBH304	Sputum	Neuro-ICU	15	KL19	–	>128 (R)	32 (R)	32 (R)	64 (R)	8 (I)	<1 (S)	64 (R)	2 (S)	<1 (S)	256 (R)	2 (S)	>64 (R)	0.25 (S)	256 (R)	1 (S)
SBH396	Sputum	EICU	15	KL19	–	>128 (R)	32 (R)	>32 (R)	>128 (R)	8 (I)	2 (S)	128 (R)	1 (S)	8 (S)	8 (R)	>256 (R)	2 (R)	0.25 (S)	>512 (R)	8 (R)
SBH397	Sputum	Neuro-ICU	15	KL19	–	>128 (R)	16 (R)	32 (R)	64 (R)	16 (R)	<1 (S)	>128 (R)	2 (S)	>128 (R)	256 (R)	8 (S)	>64 (R)	16 (R)	64 (S)	16 (R)
SBH398	Sputum	Neuro-ICU	15	KL19	–	>128 (R)	16 (R)	>32 (R)	>128 (R)	8 (I)	2 (S)	128 (R)	1 (S)	8 (S)	4 (S)	>256 (R)	>64 (R)	0.5 (S)	>512 (R)	16 (R)
SBH399	Sputum	Neuro-ICU	15	KL19	–	>128 (R)	16 (R)	>32 (R)	>128 (R)	4 (S)	2 (S)	64 (R)	1 (S)	8 (S)	4 (S)	>256 (R)	2 (R)	2 (S)	>512 (R)	16 (R)
SBH400	Sputum	Neurology	15	KL19	–	>128 (R)	16 (R)	32 (R)	128 (R)	16 (R)	<1 (S)	128 (R)	1 (S)	32 (R)	256 (R)	16 (S)	>64 (R)	16 (R)	64 (S)	16 (R)
SBH401	Urine	Neuro-ICU	15	KL19	–	>128 (R)	16 (R)	>32 (R)	>128 (R)	16 (R)	<1 (S)	>128 (R)	2 (S)	8 (S)	4 (S)	>256 (R)	>64 (R)	4 (S)	>512 (R)	16 (R)

AMP, ampicillin; CTX, cefotaxime; MEM, meropenem; GEN, gentamicin; AMK, amikacin; STR, streptomycin; TET, tetracycline; TIL, tigecycline; CHL, chloramphenicol; FFC, florfenicol; NAL, nalidixic acid; CIP, ciprofloxacin; CL, colistin; FOS, fosfomycin; SXT, trimethoprim-sulfamethoxazole.

MIC, minimum inhibitory concentration; S, sensitive; I, intermediate; R, resistant.

Neuro-ICU, Neurological Intensive Care Unit; EICU, Emergency Intensive Care Unit.

Antimicrobial susceptibility testing demonstrated uniform resistance to ampicillin, cefotaxime, meropenem, gentamicin, tetracycline, tigecycline, and colistin, while all isolates remained susceptible to amikacin and streptomycin. Notably, isolates SBH397 and SBH400 exhibited broader resistance spectra, including resistance to chloramphenicol, florfenicol, ciprofloxacin, and fosfomycin. Collectively, these findings demonstrated that all isolates exhibited an extensively drug-resistance (XDR) phenotype, thereby severely limiting available therapeutic options (**Table 1**).

### 3.2 Virulence assessment in the *G. mellonella* infection model

**Figure 1:**
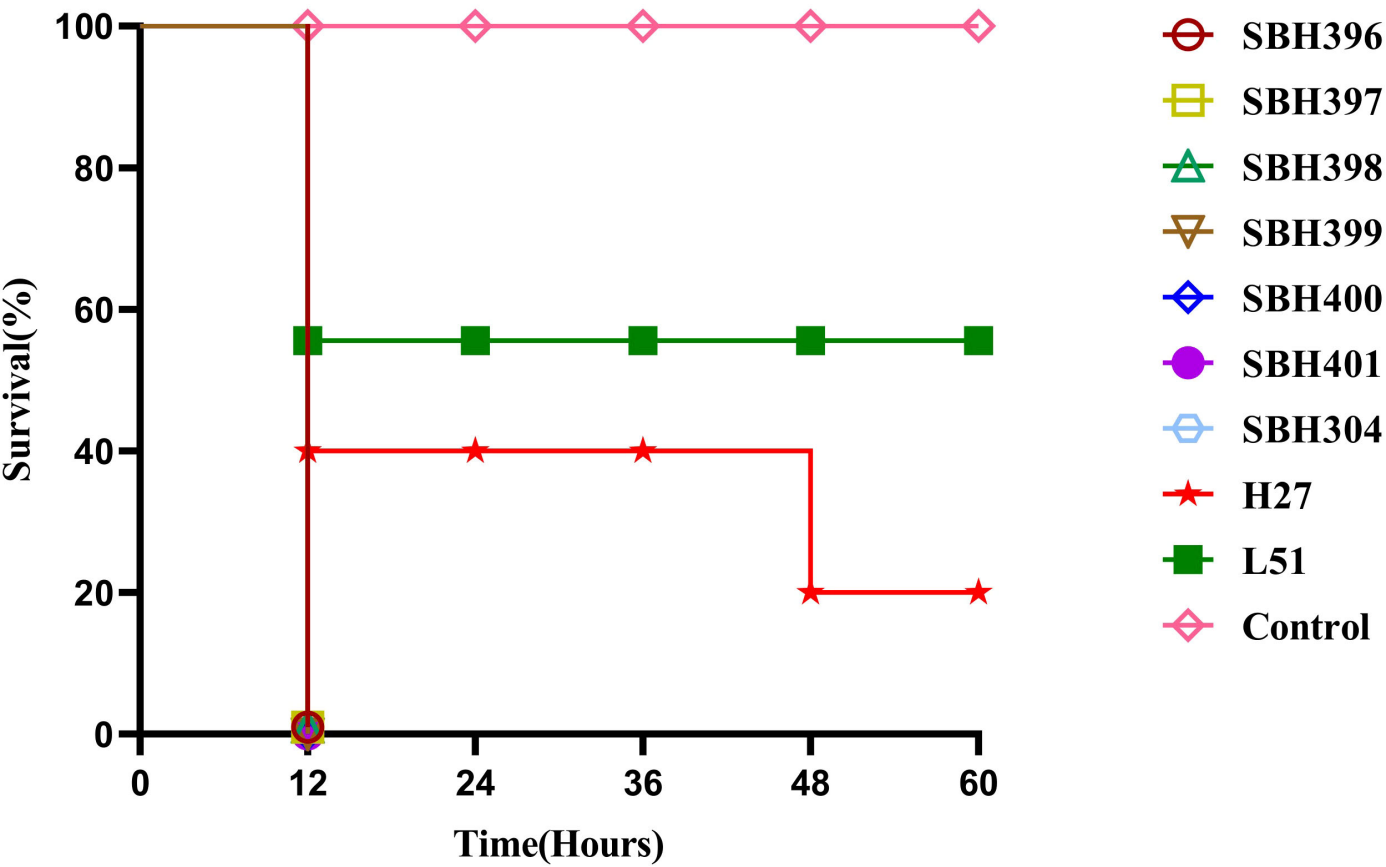
Virulence assessment of carbapenem-resistant *K. pneumoniae* isolates using the *G. mellonella* infection model. Survival curves of *G. mellonella* larvae infected with seven carbapenem-resistant *K. pneumoniae* clinical isolates (SBH304-SBH401), a hypervirulent reference strain (H27), a low-virulence strain (L51), and a PBS control. Larval survival was monitored for 60 hours post-infection. Each infection assay was performed independently three times, and the survival curves shown represent data from one representative experiment, as consistent trends were observed among replicates.

The virulence potential of the isolates was evaluated using the *G. mellonella* larvae infection model. All seven isolates caused rapid larval mortality within 12 hours post-infection, indicating a markedly elevated virulence phenotype. In comparison, the hypervirulent reference strain H27 caused 60% mortality within the same timeframe, whereas the low-virulence strain L51 induced only 10% mortality after 48 hours. These results demonstrate that the clinical isolates exhibit a high virulence potential in the infection model (**Figure 1**).

### 3.3 Genomic characterization and *bla*_KPC-2_ genetic context

Whole-genome sequencing revealed that all seven *K. pneumoniae* isolates belonged to sequence type ST15 and capsular type KL19 (**Table 2**). Isolates SBH396, SBH397, SBH399, SBH400, and SBH401 displayed highly similar genomic architectures, each comprising a single chromosome and three plasmids. In contrast, SBH398 harbored four plasmids, while SBH304 carried five. The plasmid sizes among all isolates ranged from approximately 80 to 160 kb. The *bla*_KPC-2_ gene was located on single-replicon repB(R1701) plasmids in isolates SBH304, SBH397, SBH398, and SBH399, whereas isolates SBH396, SBH400, and SBH401 harbored *bla*_KPC-2_ on multi-replicon hybrid plasmids containing IncFIB(K), IncFII(K), and repB(R1701).

**Table 2:** Genomic characteristics of *bla*_KPC-2_-carrying *K. pneumoniae* isolates.

Strain / Component	Size (bp)	GC content (%)	ST	Resistance genes	Plasmid replicon	Accession NO.
SBH304			15			CNS1384837
chromosome	5346882	57.4%		*aac(3)-IId*, *bla*_SHV-106_,*bla*_SHV-28_, *fosA6*, *oqxAB*	–	
pSBH304_1	140054	52.3%		*bla*_CTX-M-15_, *bla*_TEM-1B_	IncFIB(K)	
pSBH304_2	94044	53.1%		*bla*_KPC-2_	repB(R1701)	
pSBH304_3	76150	53.7%		*aac(6’)-Ib-cr*, *bla*_LAP-2_, *bla*_OXA-1_, *qnrS1*, *tet*(A)	IncFII(pSDP9R)	
pSBH304_4	67356	51.2%		*erm*(B)	IncFII	
pSBH304_5	18596	54.4%		*-*	ColRNAI	
SBH396			15			CNS1384838
chromosome	5467372	57.2%		*aac(3)-IId*, *bla*_SHV-106_, *bla*_SHV-28_, *fosA6*, *oqxAB*	–	
pSBH396_1	303831	52.7%		*aac(6’)-Ib-cr*, *bla*_KPC-2_, *bla*_C TX-M-15_, *bla*_TEM-1B_, *bla*_OXA-1_	IncFIB(K), IncFII(K), repB(R1701)	
pSBH396_2	74345	53.8%		*bla*_LAP-2_, *qnrS1*	IncFII(pSDP9R)	
pSBH396_3	22974	55.6%		–	ColRNAI	
SBH397			15			CNS1384839
chromosome	5467359	57.2%		*aac(3)-IId*, *bla*_SHV-106_, *bla*_SHV-28_, *fosA6*, *oqxAB*	–	
pSBH397_1	95965	53.2%		*bla*_KPC-2_	repB(R1701)	
pSBH397_2	86847	53.2%		*aac(6’)-Ib-cr*, *bla*_CTX-M-15_, *bla*_TEM-1B_, *bla*_LAP-2_, *bla*_OXA-1_, *qnrS1*,*tet*(A)	IncFII(pSDP9R)	
pSBH397_3	11970	55.6%		–	ColRNAI	
SBH398			15			CNS1384840
chromosome	5467294	57.2%		*aac(3)-IId*, *bla*_SHV-106_, *bla*_SHV-28_, *fosA6*, *oqxAB*	–	
pSBH398_1	204539	52.5%		*aac(6’)-Ib-cr*, *bla*_TEM-1B_, *bla*_OXA-1_, *tet*(A)	IncFIB(K), IncFII(K)	
pSBH398_2	130054	53.5%		*bla*_KPC-2_	repB(R1701)	
pSBH398_3	86844	53.2%		*aac(6’)-Ib-cr*, *bla*_CTX-M-15_, *bla*_TEM-1B_, *bla*_LAP-2_, *bla*_OXA-1_, *qnrS1*, *tet*(A)	IncFII(pSDP9R)	
pSBH398_4	11970	55.6%		–	ColRNAI	
SBH399			15			CNS1384841
chromosome	5467256	57.2%		*aac(3)-IId*, *bla*_SHV-106_, *bla*_SHV-28_, *fosA6*, *oqxAB*	–	
pSBH399_1	95966	53.2%		*bla*_KPC-2_	repB(R1701)	
pSBH399_2	86847	53.2%		*aac(6’)-Ib-cr*, *bla*_CTX-M-15_, *bla*_TEM-1B_, *bla*_LAP-2_, *bla*_OXA-1_, *qnrS1*, *tet*(A)	IncFII(pSDP9R)	
pSBH399_3	11970	55.6%		*-*	ColRNAI	
SBH400			15			CNS1384842
chromosome	5467922	57.2%		*aac(3)-IId*, *bla*_SHV-106_, *bla*_SHV-28_, *fosA6*, *oqxAB*	–	
pSBH400_1	300456	52.7%		*aac(6’)-Ib-cr*, *bla*_KPC-2_, *bla*_TEM-1B_, *bla*_OXA-1_	IncFIB(K), IncFII(K), repB(R1701)	
pSBH400_2	86832	53.2%		*aac(6’)-Ib-cr*, *bla*_CTX-M-15_, *bla*_TEM-1B_, *bla*_LAP-2_, *bla*_OXA-1_, *qnrS1*, *tet*(A)	IncFII(pSDP9R)	
pSBH400_3	11950	55.6%		–	ColRNAI	
SBH401			15			CNS1384843
chromosome	5498224	57.2%		*aac(3)-IId*, *bla*_SHV-106_, *bla*_SHV-28_, *fosA6*, *oqxAB*	–	
pSBH401_1	300512	52.7%		*aac(6’)-Ib-cr*, *bla*_KPC-2_, *bla*_TEM-1B_, *bla*_OXA-1_, *tet*(A)	IncFIB(K), IncFII(K), repB(R1701)	
pSBH401_2	86838	53.2%		*aac(6’)-Ib-cr*, *bla*_CTX-M-15_, *bla*_TEM-1B_, *bla*_LAP-2_, *bla*_OXA-1_, *qnrS1*, *tet*(A)	IncFII(pSDP9R)	
pSBH401_3	11947	55.6%		–	ColRNAI	

All seven isolates carried a broad spectrum of antimicrobial resistance genes distributed across both chromosomal and plasmid backbones. Chromosomal resistance determinants included *aac(3)-IId*, *bla*_SHV-106_, *blaSHV-28*, *fosA*, and the efflux pump operon *oqxAB*. Plasmid-borne resistance genes comprised *bla*_CTX-M-15_, *bla*_TEM-1B_, *bla*_OXA-1_, *bla*_LAP-2_, *aac(6′)-Ib-cr*, *qnrS1*, and *tet*(A). Notably, *erm*(B) was detected exclusively on plasmid pSBH304_4.

Virulence associated gene analysis based on the VFDB revealed that most identified virulence related genes were located on chromosome (**Supplementary Table 1**). All isolates carried a conserved set of adhesion and biofilm associated genes (*fimABCDEFGHIK*, *mrkABCDFHIJ*), together with multiple iron acquisition systems, including *iutA*, *entABCDEFS*, *fepABCDG*, and the yersiniabactin cluster (f*yuA*, *irp1/2*, *ybtAEPQSTUX*). However, genes associated with capsule biosynthesis and regulation varied among isolates, *rcsB* was detected in all isolates, *rcsA* only in SBH304, and *rmpA* in SBH304 and SBH398. These results indicate that ST15-KL19 isolates possess several conserved virulence-associated genes. However, the absence of a complete set of classical hypervirulence markers suggests that these strains should be considered as having elevated virulence potential rather than being classified as classical hypervirulent *K. pneumoniae*.

**Figure 2:**
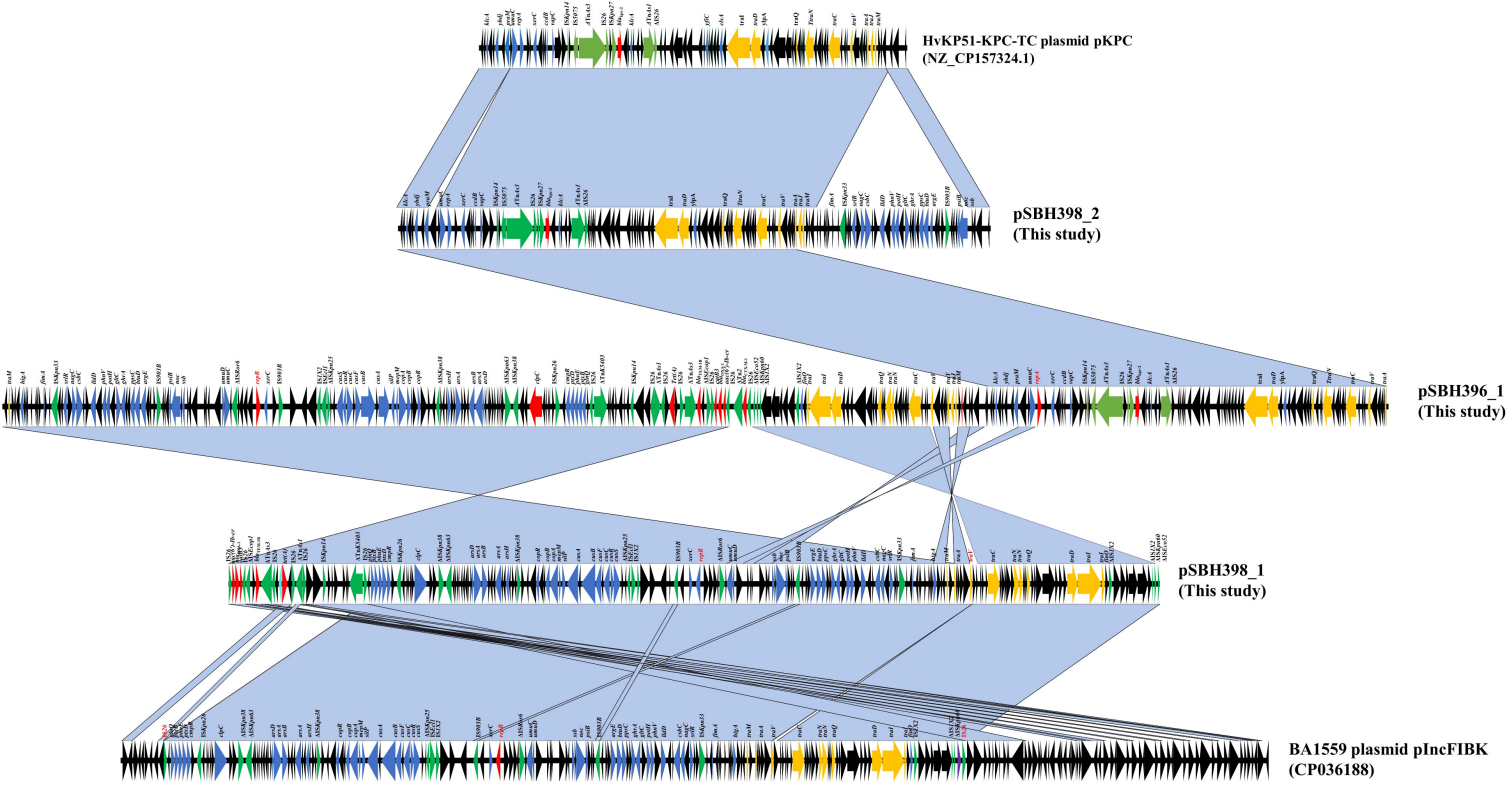
Linear genomic comparison of hybrid plasmid pSBH396_1, pSBH400_1, pSBH401_1. Arrows represent annotated open reading frames (ORFs), color-coded by functional category and oriented by transcriptional direction. Grey shaded regions indicate nucleotide sequence identity ≥90%. The symbol Δ indicates truncated genes.

**Figure 3:**
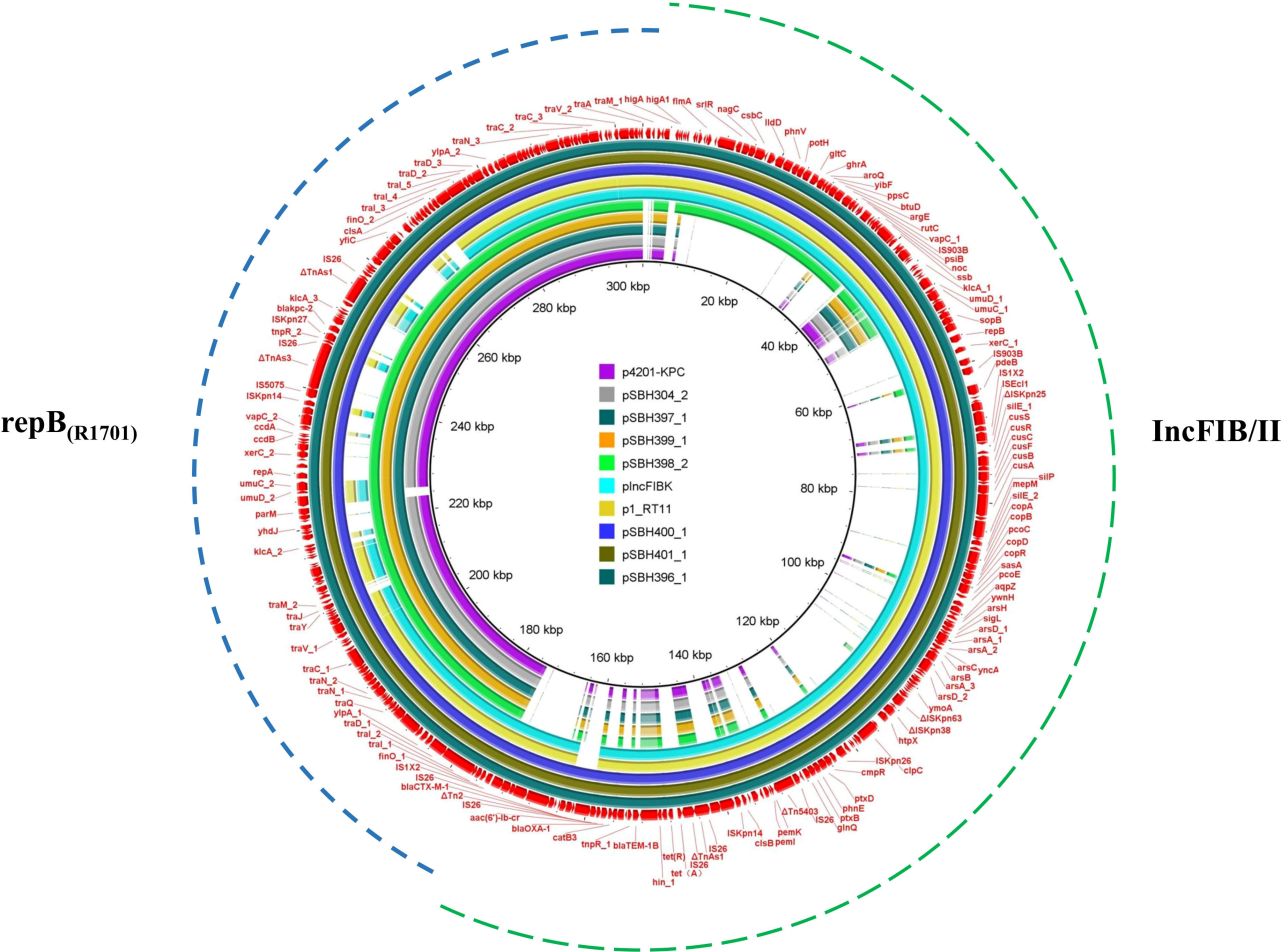
Circular alignment of hybrid plasmids and their putative ancestral backbones. Circular BLAST alignment showing hybrid plasmids pSBH396_1, pSBH400_1, and pSBH401_1, which were reconstructed from the fusion of multiple parental plasmids. Comparative reference plasmids p4201-KPC, pIncFIBK, and p1_RT11 from the NCBI database are shown for homology analysis. The outermost red ring represents the gene annotation of the reference plasmid pSBH396_1. Annotated coding sequences are displayed according to functional categories and transcriptional direction. The inner colored concentric rings represent the BLAST alignment results of all plasmids included in the comparison relative to the reference plasmid, as indicated in the legend.

Comparative plasmid analysis revealed that pSBH396_1, pSBH400_1, and pSBH401_1 were complex fusion plasmids carrying multiple replicons, including IncFIB(K), IncFII(K), and repB(R1701). Structural alignments indicated that these plasmids likely originated from multi-step homologous recombination events involving three distinct parental backbones, with each contributing a unique replicon. The resulting hybrid plasmids exhibited nearly identical genetic organizations (**Figures 2**, **3**), suggesting the presence of a conserved recombinant plasmid structure within the ST15-KL19 clone.

**Figure 4:**
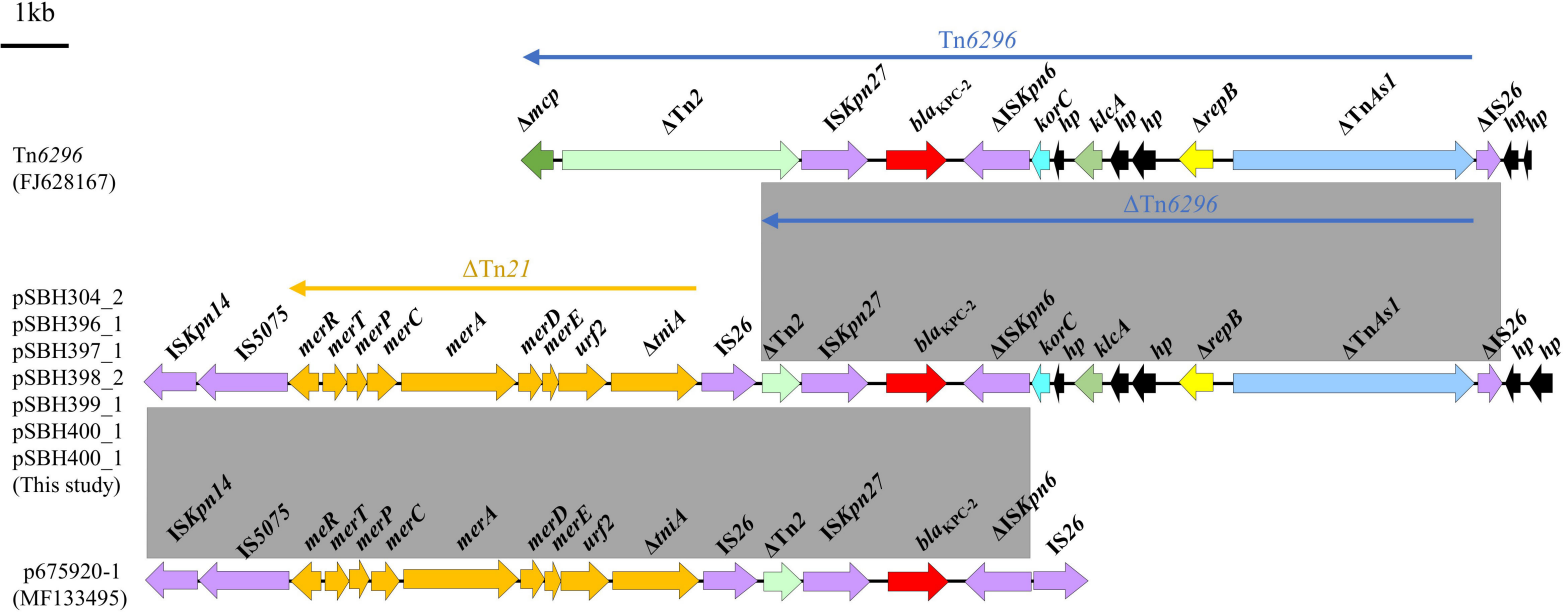
Genetic organization of the *bla*_KPC-2_ regions across representative plasmids. Genes are color-coded by function. Grey shaded areas indicate ≥90% sequence identity. The modular structure ΔTn*21*-IS*26*-ΔTn*6296*-IS*26* is conserved across isolates. Truncated genes are marked with Δ.

Further analysis of the *bla*_KPC-2_-carrying regions demonstrated that the genetic environment did not correspond to the classical Tn*4401* transposon structure (IS*Kpn7*-*bla*_KPC_-IS*Kpn6*-*tnpA*-*tnpR*). Instead, a conserved composite structure was identified among plasmids pSBH304_2, pSBH396_1, pSBH397_1, pSBH398_2, pSBH399_1, pSBH400_1, and pSBH401_1. All shared a modular configuration, designated ΔTn*21*-IS*26*-ΔTn*6296*-IS*26* (**Figure 4**). The upstream region comprised a truncated Tn*21* fragment containing IS*Kpn14*, IS*5075*, and a complete *mer* operon (*merR*, *merT*, *merP*, *merC*, *merA*, *merD*, *merE*), followed by *urf2* and Δ*tniA*. This upstream module was linked by an IS*26* insertion to a downstream ΔTn*6296* segment carrying IS*Kpn27*, *bla*_KPC-2_, ΔIS*Kpn6*, *korC*, *klcA*, and *repB*. The configuration was terminated by an additional IS*26* element at the distal end. This conserved organization supports an IS*26*-mediated recombination mechanism underlying the assembly of the *bla*_KPC-2_ platform in ST15-KL19 isolates.

### 3.4 Conjugative transfer and stability of *bla*_KPC-2_-harboring plasmids

All seven isolates successfully transferred *bla*_KPC-2_-carrying plasmids to *E. coli* C600 under the experimental conditions tested. The resulting transconjugants exhibited elevated meropenem MICs ranging from 4 mg/L to >32 mg/L (**Supplementary Table 2**).

**Figure 5:**
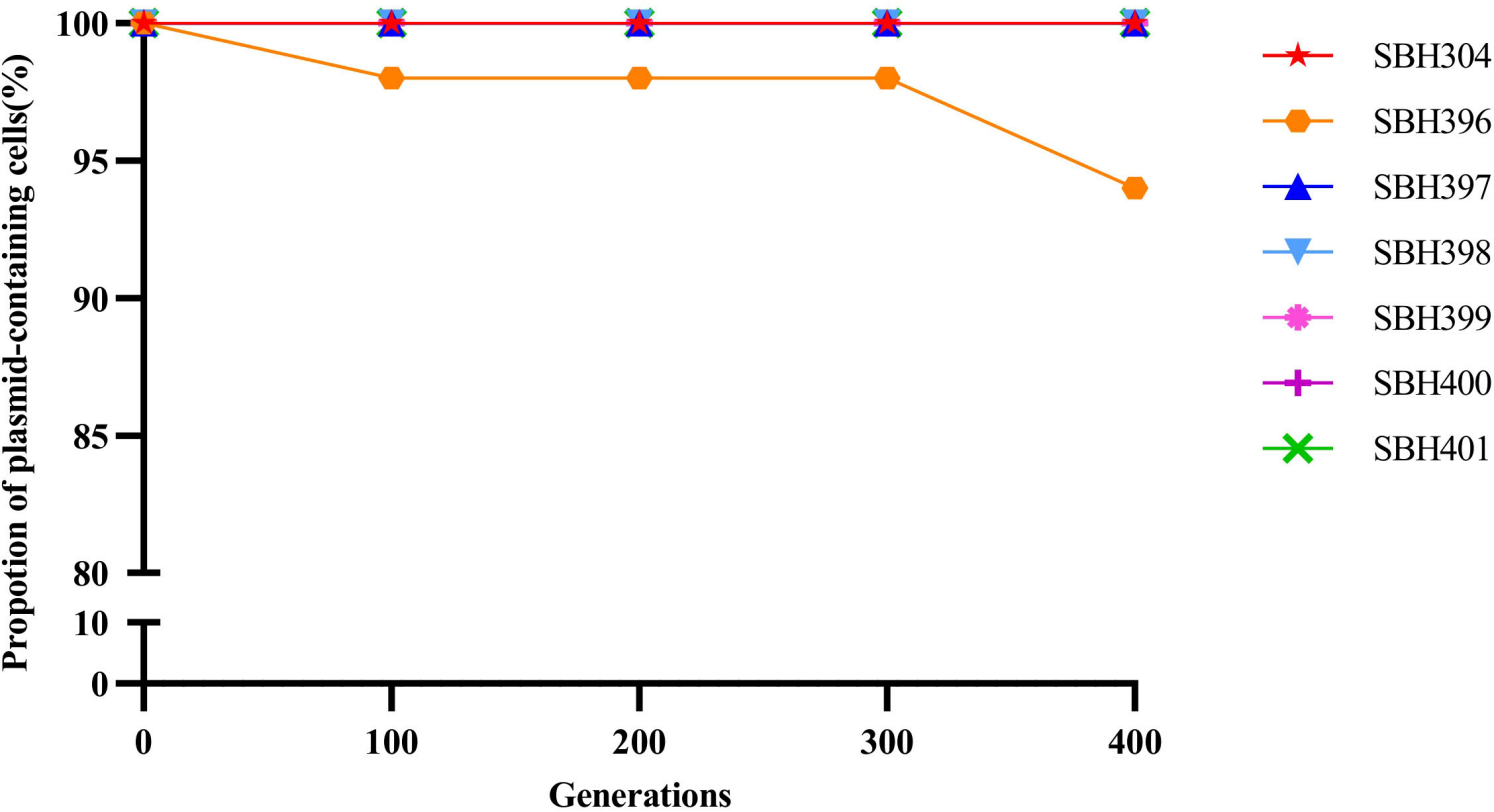
Stability of *bla*_KPC-2_-carrying plasmids during 400 generations of serial passage in antibiotic-free LB medium. Retention rates of *bla*_KPC-2_-harboring plasmids in ST15-KL19 isolates following 20 serial passages in antibiotic-free LB medium. The proportion of plasmid-containing cells was determined every 100 generations via PCR detection of *bla*_KPC-2_ in randomly selected colonies.

Plasmid stability assays further revealed that, after approximately 400 generations (corresponding to 20 consecutive passages) in antibiotic-free LB broth, more than 90% of cells retained the *bla*_KPC-2_-bearing plasmids in all tested isolates. These results indicate that the plasmids exhibit high stability and strong maintenance capacity, enabling long-term persistence and efficient horizontal dissemination even in the absence of antibiotic selective pressure (**Figure 5**).

### 3.5 Phylogenetic relationships and global dissemination of ST15-KL19 isolates

**Figure 6:**
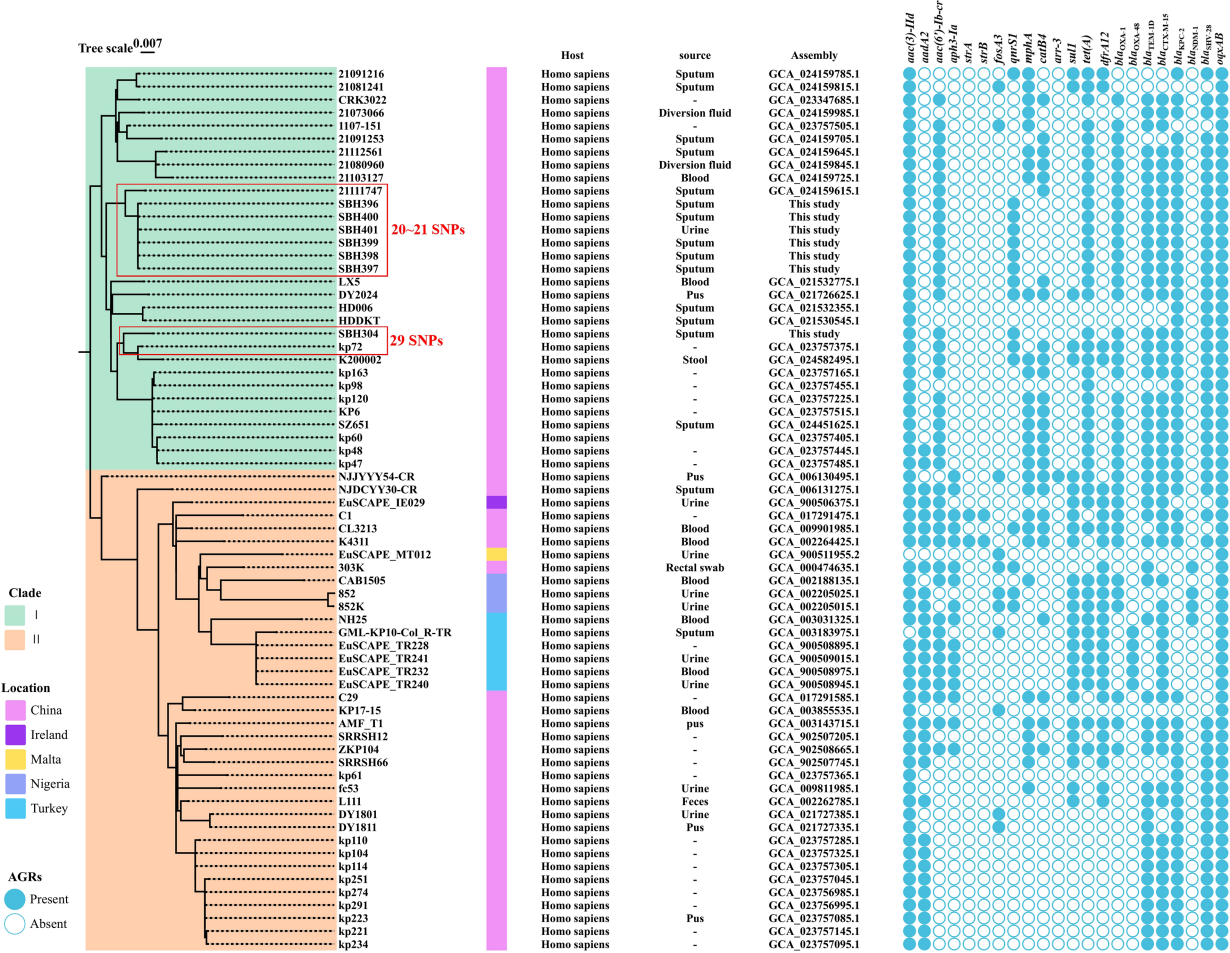
Phylogenetic analysis of global ST15-KL19 *K. pneumoniae* isolates reconstructed from core genome SNPs. Maximum-likelihood tree based on core genome SNPs from 68 ST15 isolates. Study isolates (SBH304-SBH401) are highlighted and clustered within Clade I, predominantly composed of Chinese clinical isolates. Geographic origin and antimicrobial resistance gene profiles are annotated. Tree scale represents SNP distance.

To investigate the genetic relatedness and global dissemination patterns of ST15-KL19 *K. pneumoniae*, genome assemblies annotated as ST15-KL19 were retrieved from the NCBI database. After excluding isolates lacking clear metadata regarding isolation source or country, more than 300 eligible genomes remained. From these, 68 isolates were selected for core-genome single-nucleotide polymorphism (SNP) analysis, taking into account their country of origin to ensure geographic representation while maintaining computational feasibility and minimizing redundancy. Core-genome SNP analysis was performed using ParSNP, and a maximum-likelihood phylogenetic tree was reconstructed. A total of 4,873 conserved core SNPs were identified. Based on well-supported branching patterns in the maximum-likelihood phylogenetic tree and distinct SNP clustering among the isolates, two major clades were defined and designated as Clade I and Clade II (**Figure 6**). Clade I was predominantly composed of Chinese isolates. In contrast, Clade II comprised isolates from multiple countries, including China, Ireland, Malta, Turkey, and Nigeria, displaying greater geographic diversity and genetic heterogeneity.

All seven isolates from this study were grouped within Clade I, exhibiting minimal genetic divergence with pairwise SNP distances ranging from 20 to 21. These isolates were most closely related to *K. pneumoniae* strain 211111747, which was recovered from a sputum sample in China, and shared highly similar resistance gene profiles, including the presence of *bla*_KPC-2_. Among them, SBH304 showed the highest similarity to strain kp72, differing by only 29 SNPs. The high genetic homogeneity and conserved resistance profiles observed among Clade I isolates suggest that this lineage has undergone regional clonal dissemination within China.

## 4 Discussion

This study reports a multidrug-resistant *K. pneumoniae* ST15-KL19 clone carrying *bla*_KPC-2_ recovered from hospitalized patients in eastern China. This lineage is consistent with the resistance-virulence convergence increasingly observed in globally disseminated sequence types such as ST11, ST147, and ST307 (Rodrigues et al., 2023; Peirano et al., 2020; Feng et al., 2023). These findings support previous reports indicating that ST15 lineages can acquire both antimicrobial resistance and virulence determinants. Nationwide surveillance in China has documented the dissemination of carbapenem-resistant ST11 and ST15 lineages with elevated virulence potential (Yang et al., 2022).

The coexistence of *bla*_KPC-2_ with additional resistance determinants, including *bla*_CTX-M-15_, *bla*_TEM-1B_, *aac(6′)-Ib-cr*, *qnrS1*, and *tet(A)*, reflects horizontal gene transfer shaping this lineage. The associated plasmids harbored hybrid replicon backbones combining IncFIB(K), IncFII(K), and repB(R1701) and exhibited a high degree of sequence conservation, consistent with similar architectures reported in clinical isolates from various geographic regions (Rodrigues et al., 2023; Wang et al., 2022; Jin et al., 2021). Functionally, these plasmids demonstrated efficient conjugative transfer and high stability during serial passaging, with more than 90% of host cells retaining *bla*_KPC-2_-carrying plasmids in the absence of antibiotic pressure. Such stability may facilitate sustained dissemination within healthcare settings.

Notably, the genetic environment of *bla*_KPC-2_ in these isolates did not correspond to the classical Tn*4401* structure widely reported in KPC-producing *K. pneumoniae*. Instead, *bla*_KPC-2_ was embedded within an IS*26*-mediated composite structure. This distinct configuration represents an alternative genetic organization of *bla*_KPC-2_ and reflects the modular rearrangement of resistance elements that may facilitate its dissemination and stabilization in diverse plasmid backbones.

In addition to antimicrobial resistance, the ST15-KL19 isolates carried multiple virulence-associated determinants related to capsule regulation, adhesion, iron acquisition and biofilm formation. Biofilm-associated genes, including fimbrial operons (*fim*, *mrk*) and other surface-attachment factors, were identified at the genomic level. However, biofilm production was not experimentally evaluated in this study, and therefore functional conclusions regarding biofilm-forming capacity cannot be directly established. Although a complete set of classical hypervirulence markers was not identified, the presence of selected virulence-associated determinants, together with the high mortality observed in the *G. mellonella* model, indicates elevated virulence potential in this experimental system rather than classical hypervirulence. Similar convergence between *bla*_KPC-2_ and virulence-associated loci has been reported in ST11 and ST15 backgrounds (Tian et al., 2022; Jin et al., 2021), suggesting that plasmid acquisition and recombination contribute to the evolution of high-risk clones.

Core-genome SNP analysis revealed close relatedness among the seven isolates (20–21 SNP differences), consistent with recent clonal expansion. Phylogenetic reconstruction positioned these isolates within a predominantly Chinese clade, supporting regional dissemination.

Clinically, resistance to carbapenems and last-resort agents such as tigecycline and colistin limits available therapeutic options. Although *tet*(A) and the chromosomal efflux system *oqxAB* were identified, no acquired *tet*(X) or *mcr* genes and no mutations in major colistin resistance loci (*mgrB*, *pmrA/B*, *phoP/Q*) were detected, consistent with observations in other *K. pneumoniae* lineages (Poirel et al., 2017). These findings highlight the complexity of genotype-phenotype relationships in antimicrobial resistance.

The co-localization of antimicrobial resistance determinants, virulence-associated determinants, and stable conjugative plasmids highlights the epidemiological relevance of ST15-KL19. Recent clinical and experimental studies have shown that carbapenem-resistant *K. pneumoniae* strains with elevated virulence potential are associated with severe disease and limited therapeutic options. The observed plasmid stability and dissemination capacity may contribute to sustained transmission within healthcare settings. Continuous genomic surveillance remains important for monitoring the spread of high-risk *K. pneumoniae* clones (Wyres and Holt, 2018).

Several limitations should be acknowledged. This investigation was conducted in a single tertiary-care hospital with a limited number of isolates, which may restrict generalizability. Detailed clinical outcome data were not available, precluding direct correlation between genomic features and disease severity. In addition, although *bla*_KPC-2_-carrying plasmids were successfully transferred, virulence-associated determinants were predominantly chromosomally encoded. Therefore, the virulence potential of transconjugant strains was not evaluated. Moreover, although the *G. mellonella* model provides preliminary insights into virulence potential, validation in mammalian infection models would further clarify clinical relevance.

## 5 Conclusion

This study describes a multidrug-resistant *K. pneumoniae* ST15-KL19 clone carrying *bla*_KPC-2_ in eastern China, characterized by the convergence of antimicrobial resistance and elevated virulence potential. Genomic and phenotypic analyses demonstrated the coexistence of multiple resistance and virulence-associated determinants supported by stable hybrid plasmids, which may facilitate persistence and dissemination within healthcare settings. These findings underscore the epidemiological relevance of ST15-KL19 and highlight the importance of continued genomic surveillance to better understand the spread of high-risk *K. pneumoniae* clones.

## Data Availability

The datasets presented in this study can be found in online repositories. The names of the repository/repositories and accession number(s) can be found in the article/**Supplementary Material**.
