# Supplementary material for: Emergence of a carbapenem-resistant Klebsiella pneumoniae ST15-KL19 clone with elevated virulence potential in a tertiary hospital in China
Source: Front Cell Infect Microbiol. 2026 Apr 7;16:1745492. doi: 10.3389/fcimb.2026.1745492 (PMC13096044; doi:10.3389/fcimb.2026.1745492)
Supplement: Supplementary file 2 [file Table2.docx]

Table S2. MIC of *K. pneumoniae* isolates carrying *bla*_KPC-2_ and their transconjugants against meropenem.

| Strain | Species | MIC (mg/L) |  |
| --- | --- | --- | --- |
|  |  | Meropenem | |
| Donor SBH304 | *K. pneumoniae* | 32 | |
| Donor SBH396 | *K. pneumoniae* | >32 | |
| Donor SBH397 | *K. pneumoniae* | 32 | |
| Donor SBH398 | *K. pneumoniae* | >32 | |
| Donor SBH399 | *K. pneumoniae* | >32 | |
| Donor SBH400 | *K. pneumoniae* | 32 | |
| Donor SBH401 | *K. pneumoniae* | >32 | |
| Transconjugant J-SBH304 | *E. coli* | 16 | |
| Transconjugant J-SBH396 | *E. coli* | 32 | |
| Transconjugant J-SBH397 | *E. coli* | 32 | |
| Transconjugant J-SBH398 | *E. coli* | 8 | |
| Transconjugant J-SBH399 | *E. coli* | 4 | |
| Transconjugant J-SBH400 | *E. coli* | >32 | |
| Transconjugant J-SBH401 | *E. coli* | 32 | |
| Recipient C600 | *E. coli* | 0.015 | |
